# Knowledge, Attitudes, and Behaviors Regarding Chemical Exposure among a Population Sample of Reproductive-Aged Women

**DOI:** 10.3390/ijerph19053015

**Published:** 2022-03-04

**Authors:** Isabel J. Ricke, Ashley Oglesby, Grace R. Lyden, Emily S. Barrett, Stacey Moe, Ruby H. N. Nguyen

**Affiliations:** 1 Division of Epidemiology & Community Health, School of Public Health, University of Minnesota, Minneapolis, MN 55454, USA; ricke201@umn.edu (I.J.R.); ogles024@umn.edu (A.O.); gerl0056@umn.edu (S.M.); 2 Division of Biostatistics, School of Public Health, University of Minnesota, Minneapolis, MN 55454, USA; lyden017@umn.edu; 3 Department of Biostatistics and Epidemiology, Rutgers School of Public Health, Piscataway, NJ 08854, USA; emily.barrett@eohsi.rutgers.edu

**Keywords:** phthalate, BPA, pre-conception, women, knowledge

## Abstract

We examined the knowledge and attitudes of reproductive-age women toward environmental chemicals and determined how these affect consumer behaviors. At the 2018 Minnesota State Fair, a large community sample of reproductive-age women was recruited to complete a survey on environmental health attitudes and behaviors. Descriptive statistics, chi-square tests, and logistic regression models were used to characterize current attitudes about chemicals. Multivariable logistic regression models examined how sociodemographic characteristics predict knowledge, attitudes, and consumer behaviors. A total of 871 women completed the survey; 74% strongly agreed that chemicals in the environment are dangerous, and 44% of women reported having heard of phthalates, while only 29% reported always practicing at least one environmentally healthy behavior (such as consuming food and beverages from safe plastics). Older age (35–39 versus 18–24: aOR 2.3, 95% CI 1.3, 4.3; 40–44 versus 18–24; aOR 2.0, 95% CI 1.2, 3.2) and working in a healthcare profession (aOR: 1.7, 95% CI: 1.2, 2.5) were associated with strong agreement that chemicals in the environmental are dangerous. Women who strongly agreed chemicals are dangerous were more likely to practice consumer behaviors to reduce their exposure. Interventions targeting knowledge and attitudes towards environmental chemicals could be an effective strategy for reducing harmful exposures.

## 1. Introduction

Chemicals are ubiquitous in our environment, including in food, water, and many consumer products such as household cleaners, personal-care products, and food packaging [1,2,3]. In 2021, the American College of Obstetricians and Gynecologists updated their 2013 opinion on prenatal exposure to environmental chemicals, highlighting increasing evidence that exposure to toxic environmental chemicals is associated with adverse reproductive and developmental outcomes [1,4]. The magnitude of this problem is demonstrated by the high frequency of detection of multiple chemicals in biomonitoring cohort studies in both North America and Europe [5,6,7,8,9,10]. These studies indicate there is widespread exposure of pregnant women to chemicals such as metals, organophosphates, environmental phenols, and phthalates, which are associated with preterm birth, miscarriage, low birth weight, and neurodevelopmental impairment [1,5,11]. Endocrine-disrupting chemicals, such as phthalates and Bisphenol-A (BPA), are of particular concern for reproductive health outcomes. BPA has been found to be associated with female infertility in multiple observational studies and has been associated with negative outcomes in medically assisted reproduction [12]. In addition to their association with preterm birth and low birth weight, phthalates are associated with adverse outcomes during pregnancy, such as hypertensive disease and gestational diabetes [11,13]. However, evidence suggests that women may be able to reduce their exposures to some environmental chemicals through changes to their behavior [14,15,16,17]. Diet and personal care product usage are two areas where women can modify their behavior and potentially reduce their exposures to metals, pesticides, BPA, and phthalates [1]. These changes include selecting “fragrance-free” personal care products, avoiding canned and fast foods, choosing fish low in mercury, and preparing and storing food in non-plastic containers [1,16,18]. While evidence is accumulating related to the link between environmental chemical exposures and reproductive and developmental health outcomes and the steps women can take to lower their exposure to certain chemicals, less is known about the knowledge and attitudes of reproductive-aged women on the subject.

Studies of pregnant women and women seeking fertility care show a large proportion recognize that environmental chemicals may be harmful to their health, and that they may try change their behavior to limit their exposures [19,20]. In The Infant Development and Environment Study (TIDES), a multicenter U.S. prospective pregnancy cohort, women who believed chemicals were dangerous were more likely to report that they practice healthy behaviors such as choosing organic foods, foods in safe plastics, chemical-free personal care products, and limiting fast food intake [19]. However, no differences in phthalate concentrations were observed between women who had planned their pregnancies and those who did not [21]. Although, women who used assisted reproductive technologies (ART), had significantly lower levels of diethylhexyl phthalate (DEHP) metabolites [22]. One potential explanation for this is that ART users may be pursuing lifestyle changes that can lower exposures to chemicals such as phthalates [22]. Additionally, knowledge may vary greatly by chemical. Awareness of chemicals such as lead, mercury, and pesticides may be high, while awareness of others, such as phthalates, may be much lower [20]. Prior research in this area has focused on women who were pregnant or planning to become pregnant. This study aims to fill in gaps in understanding by surveying a large community sample of reproductive-aged women at a public venue in Minnesota. This population is of particular concern because of their vulnerability to the effects of environmental exposures such as impacts on fertility, pregnancy, and fetal development. Our study objectives were to describe the knowledge and attitudes toward environmental chemicals among reproductive-aged women, and how these may impact their behaviors.

## 2. Materials and Methods

This study analyzed anonymous survey data from a convenience sample of 2018 Minnesota State Fair attendees, recruiting adult women of reproductive age, 18–44 years old. A total of 923 electronically collected surveys were started over the course of the four-day data collection period in August–September 2018; 871 (94%) surveys were ultimately completed. The Minnesota State Fair is attended by more than 2 million people, making it among the largest in the U.S. Women were recruited in the University of Minnesota Driven to Discover Research Building, a facility located on the fairgrounds that is dedicated to data collection. The survey was self-administered online, utilizing university-supported Qualtrics software (Provo, UT). A complete description of the data collection can be found elsewhere [23]. Questions used in this survey were adapted from those used in the TIDES study [19]. This study was approved by the University of Minnesota IRB Human Subjects Committee.

### 2.1. Demographic Data

For these analyses, age was categorized as “18–24”, “25–29”, “30–34”, “35–39”, and “40–44”. Race and ethnicity were dichotomized as non-Hispanic White or other than non-Hispanic White. Marital status was dichotomized as married/living as married/long-term relationship versus single, divorced, widowed, or other. Education was dichotomized as graduated college or more versus some college education or less.

### 2.2. Environmental Health Attitudes and Knowledge

General attitudes about environmental health and chemicals were assessed through two items with responses on a five-point Likert scale (strongly agree, agree, neither agree nor disagree, disagree, strongly disagree). The first item evaluated the extent to which participants agreed that “Chemicals in the environmental can pose health risks”. The second question evaluated the extent to which they agreed that “Chemicals in the environment are in so many things that they are impossible to avoid”. Other attitude items evaluated agreement with the statements: “Women planning to become pregnant are particularly at risk of any potential harms from chemicals in the environment”, “Pregnant women and their unborn babies are particularly at risk of any potential harms from chemicals in the environment”, “Pregnant women should avoid plastic food and beverage containers during pregnancy”, and “Pregnant women should avoid scented soaps, cosmetics and lotions during pregnancy”. We focused on modifiable behaviors of the most common phthalate exposures in the latter two questions. After reviewing the distribution of the responses and findings, a clear demarcation with the highest level versus the others, all questions were dichotomized for analysis as “strongly agree” versus all other responses.

Women also responded to items about their knowledge of environmental chemicals. This self-reported knowledge was assessed in four questions: self-reported knowledge of safe plastic recycling codes, correct identification of safe plastic recycling codes (correct response: 1, 2, 4, 5), awareness of phthalates and BPA (yes versus no), and whether participants think phthalates and BPA are the same (correct response: no).

### 2.3. Consumer Behaviors

Consumer behaviors related to environmental health were evaluated through seven items. Participants were asked the extent to which they agreed with two statements: “When I buy bath soap, cosmetics, and toiletries, I try to make sure they are eco-friendly, chemical-free, or environmentally friendly” and “When I buy or consume food, I try to make sure it is organic, all-natural, eco-friendly, chemical-free, or environmentally friendly”. Responses were again dichotomized as “strongly agree” versus all else.

Women were also asked how frequently they practice the following behaviors (always, usually, sometimes, rarely, or never): buying eco-friendly, chemical-free household products; consuming food and beverages from plastics that are safe; avoiding drinking from plastic bottles; checking the recycling code on a plastic bottle they are drinking from. These responses were dichotomized as “always” versus all else. Women were also asked how often they microwave, or otherwise warm up food, in a plastic container (including foods that may come in a plastic such as individual frozen meals). Based on distributions, responses to this question were dichotomized as “never” versus all other responses.

### 2.4. Statistical Analyses

Qualtrics recorded 923 opened surveys. Of these, 39 surveys were incomplete, either due to not providing consent (16 surveys) or never reaching the end-page (23 surveys), and an additional 13 lacked a response for age, resulting in a total of 871 surveys for analysis.

We summarized counts and proportions for all variables. We then performed bivariate analyses (chi-square tests and univariable logistic regression) to describe current attitudes, knowledge, and behaviors related to environmental chemicals by sociodemographic characteristics. Sociodemographic variables that were associated with the environmental health attitudes in the univariate analysis were used in multivariable logistic regression. Multivariable logistic regression models, controlling for age, relationship status, education, and work as a healthcare professional were used to examine whether sociodemographic characteristics predicted strong agreement that chemicals in the environment are dangerous or impossible to avoid, and whether strong agreement that chemicals in the environment are dangerous or impossible to avoid predicted consumer behaviors that would reduce exposure to chemicals. All analyses were performed using SAS v9.4 (SAS Institute Inc., Cary, NC, USA), and all *p*-values reported are two-tailed with an alpha level of *p* = 0.05.

## 3. Results

### 3.1. Sample Characteristics

Sociodemographic characteristics of participants are presented in Table 1. The average age of study participants was 30. The overwhelming majority were non-Hispanic white (84%), and 66% had at least a college degree. The majority of women (67%) were married or in a long-term relationship, and 26% reported working in a healthcare profession.

### 3.2. Attitudes, Knowledge, and Behavior Distributions

Most (74%) participants strongly agreed that chemicals in the environment are dangerous while 41% strongly agreed that chemicals in the environment are impossible to avoid (Table 1). Women who strongly agreed that chemicals are dangerous were more likely to agree that chemicals are also impossible to avoid (Χ^2^ = 16.7, *p* < 0.01; results not shown elsewhere).

Most women (93%) had heard of BPA, whereas only 44% of women had ever heard of phthalates and 29% believed the two chemicals to be the same. In general, women who were older, married, or living as married; were more educated, or who worked in a healthcare profession were more likely to know about phthalates (Table 1). More than half of women surveyed (52%) believed pregnant women and their unborn babies to be particularly at risk of potential harms from chemicals in the environment. However, few believed that pregnant women should avoid plastic food and beverage containers (16%) or scented soaps, cosmetics, and lotions (8%). Women who were older or who were married or living as married were more likely to believe pregnant women should avoid plastic food and beverage containers; and women who were older or who worked in a healthcare profession were more likely to believe pregnant women should avoid scented personal care products (Table 1).

Only 29% of participants reported always practicing at least one environmentally relevant behavior, such as consuming organic foods, purchasing eco-friendly/natural/chemical-free products, or avoiding eating, drinking or heating food in plastics (Table 2). The most commonly practiced behaviors were buying chemical-free or eco-friendly personal care products (19%) and avoiding warming food in plastic containers (19%). Women who strongly agreed that chemicals were dangerous were more likely to practice one of these behaviors (Χ^2^ = 19.5, *p* < 0.01; results not shown elsewhere).

### 3.3. Sociodemographic Chacteristics Associated with Attitudes

In a multivariable logistic regression model adjusted for education, older age (35–39 versus 18–24: OR 2.3, 95% CI 1.3, 4.3, *p* < 0.01; 40–44 versus 18–24; OR 2.0, 95% CI 1.2, 3.2, *p* < 0.01) and work as a healthcare professional (OR 1.7, 95% CI 1.2, 2.5, *p* < 0.01) were associated with strong agreement that chemicals in the environment are dangerous (Table 3). Women who were married or in a long-term relationship were less likely to strongly agree chemicals in the environment are dangerous (OR 0.7, 95% CI 0.5, 1, *p* = 0.04). In a multivariable logistic regression model adjusting for the same variables, age was associated with strong agreement that chemicals in the environment are impossible to avoid (35–39 versus 18–24: OR 2.3, 95% CI 1.4, 3.7, *p* < 0.01; 40–44 versus 18–24; OR 1.9, 95% CI 1.2, 2.8, *p* < 0.01).

### 3.4. Association between Attitudes and Behaviors

After adjusting for age, education, relationship status, and work as a healthcare professional, women who strongly agreed that chemicals in the environment were dangerous were more likely to buy “eco-friendly”/“chemical-free” personal care products (OR 2.7, 95% CI 1.6, 4.4, *p* < 0.01), buy “eco-friendly”/“chemical-free” household products (OR 3.9, 95% CI 1.9, 8, *p* < 0.01), buy/consume “organic”/“all-natural”/“eco-friendly”/“chemical-free” food (OR 2.0, 95% CI 1.1, 3.6, *p* = 0.03), try to consume food and beverages from safe plastics ( OR 3.5, 95% CI 1.4, 9.1, *p* < 0.01), and check recycling codes on bottles they drink from (OR 2.6, 95% CI 1.2, 5.6, *p* = 0.02) (Table 4). They were also more likely to avoid drinking from plastic bottles (OR 3.4, 95% CI 1.4, 8.2, *p* < 0.01) and warming food in plastic containers (OR 2.3, 95% CI 1.4, 3.7, *p* < 0.01). After adjusting for age, education, relationship status and work as a healthcare professional, there were no associations between strong agreement that chemicals are impossible to avoid and any behaviors.

**Table 1:** Demographics and characteristics of the reproductive-aged female survey participants (*N* = 871) attending the 2018 Minnesota State Fair in relation to knowledge and attitudes towards environmental chemicals.

		Attitudes						Knowledge				
Characteristic	Survey Participants	Strongly Agree That Chemicals in the Environment Are Dangerous ^1^	Strongly Agree That Chemicals in the Environment Are Impossible to Avoid ^1^	Women Planning to Become Pregnant Are at Risk ^1^	Pregnant Women and Their Unborn Babies Are at Risk ^1^	Pregnant Women Should Avoid Plastic Food and Beverage Containers ^1^	Pregnant Women Should Avoid Scented Soaps, Cosmetics and Lotions ^1^	Self-Reported Knowledge of Plastic Recycling Codes ^2^	Correct Identification of Safe Recycling Codes ^3^	Ever Heard of Phthalates	Ever Heard of BPA	Phthalates and BPA Are Not the Same Thing
*N* (%)	*N* = 871	646/871 (74)	364/871 (41)	368/871 (42)	450/870 (52)	136/870 (16)	69/868 (8)	541/871 (62)	5/407 (1)	384/867 (44)	814/867 (93)	253/871 (29)
Age												
18–24	272 (31)	191 (70)	95 (35)	106 (39)	136 (50)	28 (10)	11 (4)	135 (50)	2 (2)	86 (32)	254 (94)	73 (27)
25–29	185 (21)	134 (72)	68 (37)	60 (32)	78 (58)	23 (12)	15 (8)	110 (59)	1 (1)	90 (49)	174 (95)	46 (25)
30–34	139(16)	94 (68)	55 (40)	64 (46)	75 (54)	15 (11)	12 (9)	98 (71)	1 (1)	71 (51)	134 (96)	47 (34)
35–39	108 (12)	91 (84)	61 (56)	50 (46)	64 (59)	29 (27)	10 (9)	71 (66)	0	51 (48)	99 (92)	40 (37)
40–44	167 (19)	136 (81)	85 (51)	88 (53)	97 (58)	41 (25)	21 (13)	127 (76)	1 (1)	86 (52)	153 (93)	47 (28)
Chi-squared *p*-value		<0.01	<0.01	<0.01	0.02	<0.01	0.03	<0.01		<0.01	0.6	0.1
Race/ethnicity dichotomized												
Non-Hispanic White (NHW)	729 (84)	539 (74)	306 (42)	307 (42)	381 (52)	105 (14)	50 (7)	468 (64)	5 (1)	324 (45)	692 (95)	217 (30)
Race other than NHW	142 (16)	107 (75)	58 (41)	61 (43)	69 (49)	31 (22)	19 (14)	73 (51)	0	60 (43)	122 (86)	36 (25)
Chi-squared *p*-value		0.7	0.8	0.9	0.4	0.02	<0.01	<0.01		0.6	<0.01	0.3
Relationship status												
Married/Long-term relationship	581 (67)	427 (73)	257 (44)	243 (42)	296 (51)	107 (18)	51 (9)	377 (65)	4 (1)	268 (46)	548 (95)	176 (30)
Single/divorced/widowed/other	289 (33)	218 (75)	107 (37)	125 (43)	153 (53)	29 (10)	18 (6)	163 (57)	1 (1)	115 (40)	265 (92)	77 (27)
Chi-squared *p*-value		0.5	0.04	0.7	0.5	<0.01	0.2	0.01	0.6	0.08	0.1	0.3
Education												
Some college or less	295 (34)	209 (71)	117 (39)	235 (41)	292 (51)	50 (17)	26 (9)	145 (49)	2 (2)	88 (30)	262 (89)	187 (32)
Graduated college	573 (66)	434 (76)	246 (43)	132 (45)	156 (53)	85 (15)	43 (8)	393 (69)	3 (1)	294 (52)	549 (96)	65 (22)
Chi-squared *p*-value		0.1	0.4	0.3	0.6	0.4	0.5	<0.01	0.5	<0.01	<0.01	<0.01
Health professional												
Yes	227 (26)	185 (82)	101 (44)	111 (49)	132 (58)	42 (19)	25 (11)	147 (65)	1 (1)	120 (53)	220 (97)	81 (36)
No	641 (74)	459 (72)	262 (41)	256 (40)	317 (49)	93 (15)	44 (7)	393 (61)	4 (1)	263 (41)	592 (93)	171 (27)
Chi-squared *p*-value		<0.01	0.3	0.01	0.02	0.2	0.05	0.4	0.7	<0.01	0.01	0.01

Note: Denominators are not the same across all categories because of questions left blank. Percentages may not add up to 100 due to rounding. ^1^ Strongly agree versus all else. ^2^ Yes versus no/I don’t know. ^3^ ‘1, 2, 4, 5′ versus all other combinations of selections.

**Table 2:** Bivariate analyses examining consumer behaviors in relation to demographics and characteristics among reproductive-aged women at the Minnesota State Fair, 2018 (*N* = 871).

	Try to Buy “Eco-Friendly”, “Chemical Free” PCPs ^1^	Try to Buy “Eco-Friendly”, “Chemical Free” Household Products ^2^	Try to Buy/Consume “Organic”, “All-Natural”, Eco-Friendly”, “Chemical Free” Food ^1^	Try to Consume Food and Beverages from Plastics That Are Safe ^2^	Avoid Drinking from Plastic Bottles ^2^	How Often Do You Microwave, or Otherwise Warm Up, Your Food in the Plastic Container (Including Foods That may Come in a Plastic such as Individual Frozen Meals)? ^3^	How often do You Check the Recycling Code on a Plastic Bottle That Are You Drinking from? ^2^
*N* (%)	164 (19)	103 (12)	94 (11)	53 (6)	59 (7)	163 (19)	66 (8)
Age							
18–24	33 (12)	18 (7)	21 (8)	10 (4)	24 (9)	40 (15)	16 (6)
25–29	45 (24)	26 (14)	23 (12)	12 (6)	7 (4)	21 (11)	10 (5)
30–34	25 (18)	14 (10)	14 (10)	7 (5)	8 (6)	30 (22)	9 (6)
35–39	29 (27)	22 (20)	15 (14)	9 (8)	10 (9)	31 (29)	13 (12)
40–44	32 (19)	23 (14)	21 (13)	15 (9)	10 (6)	41 (25)	18 (11)
Chi-squared *p*-value	<0.01	<0.01	0.2	0.2	0.2	<0.01	0.09
Race/ethnicity dichotomized							
Non-Hispanic White	126 (17)	77 (11)	70 (10)	43 (6)	47 (6)	139 (19)	54 (7)
Race other than NHW	38 (27)	26 (18)	24 (17)	10 (7)	12 (8)	24 (17)	12 (8)
Chi-squared *p*-value	<0.01	<0.01	0.01	0.6	0.4	0.5	0.7
Relationship status							
Married/Long-term relationship	117 (20)	76 (13)	67 (12)	43 (7)	38 (7)	121 (21)	47 (8)
Single/divorced/widowed/other	46 (16)	27 (9)	27 (9)	10 (3)	21 (7)	42 (15)	19 (7)
Chi-squared *p*-value	0.1	0.1	0.3	0.02	0.7	0.02	0.4
Education							
Some college or less	51 (17)	33 (11)	29 (9)	23 (8)	23 (8)	49 (17)	31 (11)
Graduated college	112 (20)	70 (12)	65 (11)	30 (5)	36 (6)	113 (20)	35 (6)
Chi-squared *p*-value	0.4	0.7	0.5	0.1	0.4	0.3	0.02
Health professional							
Yes	50 (22)	30 (13)	36 (16)	18 (8)	16 (7)	34 (15)	21 (9)
No	113 (18)	73 (11)	57 (9)	35 (5)	42 (7)	128 (20)	44 (7)
Chi-squared *p*-value	0.1	0.5	<0.01	0.2	0.8	0.09	0.2

Note: Denominators are not the same across all categories because of questions left blank. Percentages may not add up to 100 due to rounding. ^1^ Strongly agree versus all else. ^2^ Always versus all else. ^3^ Never versus all else.

**Table 3:** Adjusted odds ratios and 95% confidence intervals for the association between sociodemographic characteristics and attitudes towards chemicals in the environment among reproductive-aged women at the Minnesota State Fair, 2018 (*N* = 867) *.

Characteristic	Strong Agreement That Chemicals in Environment Are Dangerous		Strong Agreement That Chemicals in the Environment Are Impossible to Avoid	
	OR (95% CI)	*p*-Value	OR (95% CI)	*p*-Value
Age				
18–24	1.0 (ref)	-	1.0 (ref)	-
25–29	1 (0.6, 1.5)	0.9	1 (0.7, 1.6)	0.9
30–34	0.8 (0.5, 1.4)	0.5	1.2 (0.8, 1.8)	0.5
35–39	2.3 (1.3, 4.3)	<0.01	2.3 (1.4, 3.7)	<0.01
40–44	2.0 (1.2, 3.2)	<0.01	1.9 (1.2, 2.8)	<0.01
Married/Long-term relationship (ref: single, divorced, widowed, other)	0.7 (0.5, 1)	0.04	1.1 (0.8, 1.5)	0.5
College graduate	1.4 (0.9, 2)	0.1	1 (0.8, 1.4)	0.8
Health professional	1.7 (1.2, 2.5)	<0.01	1.2 (0.8, 1.6)	0.4

* Four women were excluded due to missing data. Adjusted for all variables in the table.

**Table 4:** Adjusted odds ratios and 95% confidence intervals for the association between consumer behaviors and attitudes towards environmental chemicals among reproductive-aged women at the Minnesota State Fair, 2018.

	Strongly Agree They Try to Buy “Eco-Friendly”, “Chemical Free” PCPs		Always Try to Buy “Eco-Friendly”, “Chemical Free” Household Products		Strongly Agree They Try to Buy/Consume “Organic”, “All-Natural”, “Eco-Friendly”, “Chemical Free” Food		Always Try to Consume Food and Beverages from Plastics That Are Safe		Always Avoid Drinking from Plastic Bottles		Never Microwave, or Otherwise Warm up, Your Food in the Plastic Container		Always Check the Recycling Code on a Plastic Bottle That Are You Drinking from?	
*N*	866		866		867		867		867		866		866	
	OR (95% CI)	*p*-Value	OR (95% CI)	*p*-Value	OR (95% CI)	*p*-Value	OR (95% CI)	*p*-Value	OR (95% CI)	*p*-Value	OR (95% CI)	*p*-Value	OR (95% CI)	*p*-Value
Strong agreement that chemicals in the environment are dangerous	2.7 (1.6, 4.4)	<0.01	3.9 (1.9, 8)	<0.01	2.0 (1.1, 3.6)	0.03	3.5 (1.4, 9.1)	<0.01	3.4 (1.4, 8.2)	<0.01	2.3 (1.4, 3.7)	<0.01	2.6 (1.2, 5.6)	0.02
Strong agreement that chemicals in the environment are impossible to avoid	1.3 (0.9,1.9)	0.1	1.5 (1, 2.2)	0.08	1.1 (0.7, 1.7)	0.6	1.3 (0.8, 2.4)	0.3	1.2 (0.7, 2)	0.6	1.2 (0.8, 1.7)	0.3	1.3 (0.8, 2.2)	0.3

Note: each column represents a separate model of how attitudes predict behavior adjusted for age, education, relationship status and work as healthcare professional.

## 4. Discussion

We evaluated the attitudes and knowledge of reproductive-age women towards environmental chemicals and their associations with consumer behaviors. Nearly three quarters of the respondents strongly agreed environmental chemicals are dangerous, while less than half strongly agreed that environmental chemicals are impossible to avoid. Strong agreement that environmental chemicals are dangerous was associated with self-report of several protective behaviors, such as purchasing eco-friendly and chemical-free personal care and household products and trying to consume food and beverages from safe plastics. This is consistent with results from a similar analysis in a cohort of pregnant women [19] and suggests that if women are made aware of potential risks associated with environmental chemicals and effective strategies to limit exposures, they may take personal action to reduce their exposure.

Additionally, most women surveyed did not recognize the additional risks of exposure to environmental chemicals for women planning to become pregnant, pregnant women, and unborn babies. Few women (16%) thought that pregnant women should avoid products such as plastic food and beverage containers or scented soaps, cosmetics, and lotions. However, women who worked as health professionals were more likely to recognize the risk of environmental chemicals in these populations and know about strategies for reducing harmful exposures. Despite this increased knowledge among healthcare professionals, they were no more likely than other women surveyed to practice most exposure reducing behaviors. This finding is consistent with previous reports that found while women may recognize that chemicals can be harmful to their health, they may not know of or practice behaviors to reduce their exposures [19,20,24]. This also highlights the importance of providing education to women on not only the risks of environmental chemicals, but also specific behaviors that can reduce harmful exposures.

While almost all women surveyed had heard of BPA, less than half had heard of phthalates. Prior studies have found that awareness of endocrine-disrupting chemicals, particularly phthalates, lags knowledge of other environmental exposures such as lead, mercury, and pesticides, which women are more widely aware of and concerned about [20,24]. In recent years, there have been public awareness campaigns aimed at increasing knowledge of BPA [25,26,27]. Furthermore, in our study, those with a higher educational attainment and those who worked in a healthcare setting were more likely to have heard of it. These types of approaches used to increase public awareness of BPA may be an effective strategy to increase knowledge of other ubiquitous, but less widely known chemicals like phthalates. Public attention of BPA rapidly increased because of effective translation and communication of scientific research on the health risks, environmental sources, economic impacts, and politics of BPA. Environmental health advocates and major media sources were instrumental in increasing awareness of BPA [28]. Consequently, BPA is now more strictly regulated and has been prohibited in products such as baby bottles, sippy cups, and infant formula packaging [27]. This has also led to extensive research on the potential sources of BPA, such as its presence in materials used in medically assisted reproduction [29].

One limitation of our study is that we used a convenience sample that may not be generalizable to other populations. Our sample was overwhelming white, non-Hispanic, and highly educated, and most women were either married or in a long-term relationship. Additionally, there is the potential for misclassification in our participant’s reported attitudes. Women may have selected responses that they felt would be more socially acceptable but did not reflect their actual behaviors and beliefs. However, even with the potential for misclassification, many women were honest about their lack of knowledge about phthalates, suggesting it is reasonable to believe they were honest when answering other questions in the survey as well. Finally, we did not ask any questions regarding pregnancy or birth history, which could also influence knowledge and behaviors.

Despite these limitations, this important research adds to the limited information currently available related to women’s knowledge and attitudes towards environmental chemicals. Describing the knowledge and attitudes in a population of reproductive-age women is important as this group is particularly vulnerable to environmental exposures [1,30]. Our results suggest that while awareness of the risks related to environmental chemicals is high, behaviors to limit exposures are much less common. Additionally, awareness of the dangers of environmental chemicals was associated with increased healthy behaviors, further emphasizing the importance of interventions targeting knowledge and attitudes towards environmental chemicals as a strategy for reducing harmful exposures.

## 5. Conclusions

In conclusion, in a large sample of reproductive-age women, we observed that belief that environmental chemicals are dangerous was associated with behaviors that may limit harmful exposures to such chemicals. Individuals may be willing to change their behaviors to limit exposures to harmful chemicals if equipped with the necessary knowledge. Nearly all study participants had heard of BPA; however, other chemicals, such as phthalates, were lesser known, which represents a significant challenge. In light of the success of efforts to raise the awareness of BPA, future research should evaluate the educational campaigns aimed at increasing the awareness of lesser-known chemicals such as phthalates in reproductive-age women, which could reduce exposures prior to pregnancy.

## Data Availability

This data will be made available for reasonable data requests after data sharing agreements have been reached.
